# Variability of Terminology Used to Describe Unwanted Workplace Behaviors in Nursing: A Scoping Review

**DOI:** 10.1177/01939459251323680

**Published:** 2025-03-17

**Authors:** Krishna Lambert, Karen Francis, Kathleen Tori

**Affiliations:** 1 University of Canberra, Canberra, ACT, Australia; 2 Charles Sturt University, Wagga Wagga NSW, Australia

**Keywords:** bullying, incivility, vertical violence, horizontal violence, nursing

## Abstract

**Background:**

Unwanted workplace behaviors impact all organizations, but in the discipline of nursing, these behaviors impact both the welfare of nurses and the quality and safety of patient care. The terminology adopted to describe these behaviors varies widely, leading to confusion and inconsistency in research and practice.

**Objectives:**

This scoping review aims to explore the variability in the terminology used to describe unwanted workplace behaviors in nursing, identify the most commonly used terms, and analyze the implications of this variability for research, policy, and practice.

**Methods:**

The Population, Concept, and Context mnemonic was used to frame the review, as recommended by the Joanna Briggs Institute. A comprehensive literature search was conducted using relevant databases. Keywords used in the search included “moral harassment, nursing, definition, meaning, description,” “Shaming,” “Mobbing,” “Bullying,” “Vertical violence,” “Horizontal violence,” “Incivility,” “Microaggression,” “Lateral violence.” Boolean operators (AND, OR) were used to combine search terms appropriately.

**Results:**

The initial search yielded 299 references. Following full-text screening, 51 studies met the inclusion criteria and were included in the final review. The review revealed 13 different terms used to describe unwanted workplace behaviors.

**Conclusion:**

The review highlights a lack of consensus across the discipline. There is a call for a singular term to be applied across the field which would support policy implementation and practice.

The literature suggests that unwanted workplace behaviors in healthcare are common and directly impact recruitment, retention, and patient safety.^1^ The shortages of healthcare personnel are felt globally, and in Australia, the baseline projections show an undersupply by the year 2035 of 70,707 FTE nurses.^2^ Recent studies have demonstrated a significant correlation with unwanted workplace behaviors and turnover intention.^3,4^

Westbrook et al,^1^ in their prevalence study of unwanted workplace behaviors, reported that 93.6% of nurses recounted experiencing at least 1 episode of unprofessional behavior in the past 12 months. Thirty-eight percent reported experiencing the behaviors from co-workers, weekly or more frequently. However, it is not only the paid workforce who are experiencing these behaviors. Student nurses, who participate in workplace learning, are particularly vulnerable to unwanted workplace behaviors, despite not being considered part of the permanent workforce. Although students are not officially in their “workplace,” the literature highlights that students frequently experience such behaviors.^5^ Hallett et al^6^ described that 81% of nursing students reported experiencing nonphysical aggression while on clinical placement.

When addressing this phenomenon, many authors in this field refer to the broader term of “workplace violence.” This term describes behaviors such as verbal and physical attacks and includes patients as perpetrators as well as victims.^54-56^ Violence against patients and violence between patients and healthcare providers was outside the scope of this review.

The main issue facing researchers in the field of unwanted workplace behavior is the variation in the terms used to describe it, complicating comparisons across disciplines and countries.^7^ To address unwanted workplace behaviors and to support a sustainable nursing workforce, a consensus of terms is needed. The lack of consensus complicates efforts to clarify, measure, and address the phenomenon,^5^ which exacerbates the situation, negatively affecting the psychological welfare of healthcare staff and students often leading to financial loss in terms of workers compensation, recruitment, and retention.^2^

Terms such as *bullying*, *abuse*, *vertical* and *horizontal violence*, *harassment*, and *incivility* have been used interchangeably in scholarly literature.^7,8^ This inconsistency presents challenges for researchers striving to compare, contrast, and amalgamate findings in this field. Furthermore, this variability can impede the development of effective interventions and policies. Clear and consistent terminology is fundamental for policymaking and implementation, as variability can lead to gaps in protection and enforcement. In practice, nurses and healthcare managers may struggle to recognize and address unwanted behaviors without a shared understanding of terms.

## Methods

As recommended by the Joanna Briggs Institute (JBI), the Population, Concept and Context (PCC) mnemonic was used to frame the review.^9^ The population was defined as nurses and/or student nurses. Concept was defined as terminology used to describe unwanted workplace behaviors. The workplace, which included educational providers, was defined as the context. The PCC framework provided the foundation for the research question of “What terms are used to describe unwanted workplace behaviors in nursing?”

### Search Strategy and Inclusion Criteria

A comprehensive literature search was conducted using 4 databases: OVID, CINAHL, Medline, and EBSCO Plus. As the phenomena first appeared in nursing literature in the 1980s with Meissner coining the phrase “nurses eat their young” in 1986, a date range of 1984-2024 was applied to the search. Search terms included “moral harassment,” “nursing,” definition, meaning, description, “shaming,” “mobbing,” “bullying,” “vertical violence,” “horizontal violence,” “incivility,” “microaggression,” and “lateral violence.” Boolean operators (AND, OR) were used to combine search terms appropriately. Articles were included in the search if they were published between January 1, 1984, and January 1, 2024, written in English, and they were original research articles. Studies were excluded if they did not relate to health, they were nonresearch papers, they did not include a definition, or they were published in a language other than English.

### Screening Process

The literature selection process was managed using Covidence (Veritas Health Innovation, Melbourne, Australia), an online software platform designed to streamline the production of systematic reviews. There were 3 steps involved in the study selection process. The first step was to import all references identified from the database searches into Covidence, where duplicates were automatically removed. The next step was to screen titles and abstracts of the imported studies. Applying the inclusion and exclusion criteria, 2 independent reviewers screened the titles and abstracts of all imported references. Any discrepancies between reviewers were resolved through consultation with a third reviewer. The final step was to undertake a full-text review. Studies that met the inclusion criteria during the title and abstract screening were retrieved for full-text review. Full-text articles were independently assessed by the same reviewers to determine their eligibility. A third reviewer resolved any disagreements. The interrater reliability analysis produced a Cohen’s kappa value of 0.46, indicating a moderate agreement between raters.

### Data Extraction and Analysis

A standardized data extraction form was developed and pilot-tested on a sample of included studies to ensure consistency and comprehensiveness. Data extracted from each study included the study characteristics (author, year, country), aim of the study, study design, type of participant, characteristics of terms used, and definitions. A textual narrative synthesis was used. The goal of a textual narrative synthesis is to provide a coherent summary of the findings while highlighting patterns, relationships, and variations across studies.^10^

## Results

**Figure 1.**
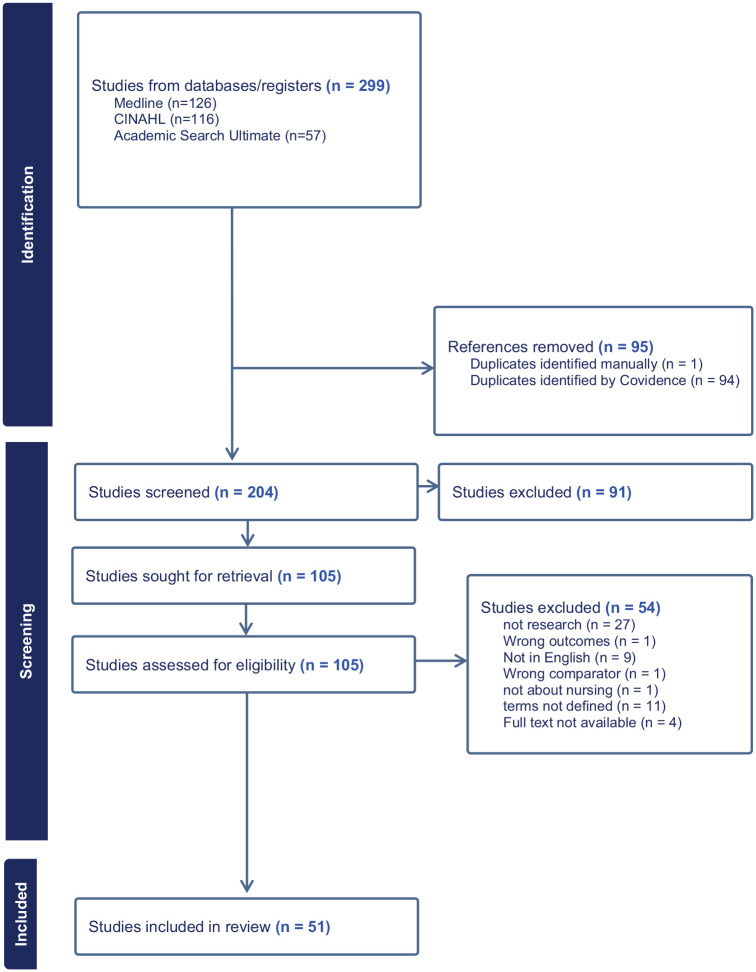
Study Screening Process.

The initial search produced 299 references which were imported into Covidence. After the removal of 95 duplicates, 204 references were screened based on titles and abstracts. Of these, 102 articles were retrieved for full-text review. Following the full-text screening, 51 studies met the inclusion criteria and were included in the final review (see Figure 1).

### Study Characteristics

The included studies varied in their design, target populations, and terminology used. The majority of the studies were conducted in the United States (*n* = 24), Canada (*n* = 4), Iran (*n* = 3), Korea (*n* = 3), Australia (*n* = 3), Turkey (*n* = 2), Brazil (*n* = 2), with one study each in South Africa, New Zealand, India, Italy, the United Kingdom, China, Lithuania, Norway, Sweden, and Switzerland. The types of studies were mainly qualitative in design such as descriptive and concept analysis. Quantitative designs were mainly cross-sectional studies. Of the articles that disclosed the target population, 30 studies recruited clinicians as participants and 6 focused on student nurses.

### Terminology and Definitions

Thirteen different terms used to describe unwanted workplace behaviors were identified. The most frequently encountered terms in the literature were bullying and incivility (Table 1). The definitions of these terms overlapped and were used interchangeably (Table 2). Many of the definitions of bullying included the term “incivility”^11^ in the description of behaviors, while the definition of incivility also included the descriptors of bullying.^12^ Many of the articles included more than one definition of terms.

**Table 1.** Frequency of terms.

Term	*n*
Bullying	16
Incivility	16
Workplace violence	5
Verbal violence	2
Moral harassment	2
Shaming	2
Mobbing	1
Humiliation	1
Vertical violence	1
Contrapower harassment	1
Intimidation	1
Negative workplace behavior	1
Horizontal violence	1

**Table 2.** Definitions of terms.

Author	Date	Country	Term	Definition Used
Hartin et al ^26^	2019	Australia	Bullying	No universally accepted definition of bullying in the nursing literature for research conducted in Australia.
O’Flynn-Magee et al^24^	2020	Canada	Bullying	Any inappropriate conduct or comment directed by one worker or student to another, that the worker knew, or should have known, would result in the worker or student feeling humiliated or intimidated. This definition excludes any reasonable actions taken by an employer or supervisor in relation to managing and directing workers, students, or the workplace.
Leong et al^11^	2016	China	Bullying	An activity that encompasses behaviors such as horizontal or lateral violence, oppressed group behavior, incivility, harassment, counterproductive behavior, and aggression.
Lee et al ^25^	2022	Korea	Bullying	Verbal violence, work-related violence, and physical violence.
Park and Kang^12^	2017	Korea	Bullying	Refers to harassing, offending, or socially excluding someone. To be labeled as bullying (or mobbing), the behavior must occur repeatedly and regularly (e.g., weekly) over an extended period of time (e.g., approximately six months).
Malinauskiene et al^22^	2011	Lithuania	Bullying	Describes a situation in which an individual is subjected to social isolation or exclusion, their work and efforts are devalued, they are threatened, derogatory comments are made about them, or other negative behaviors are directed at them with the intent to torment, wear down, or frustrate the target.
Reknes et al^19^	2014	Norway	Bullying	Refers to excessive exposure to unwanted and potentially harmful behaviors from other members of the organization over a prolonged period within the workplace.
Strandmark et al^23^	2019	Sweden	Bullying	Is a psychosocial phenomenon.
Katrinli et al^15^	2010	Turkey	Bullying	Any type of repetitive verbal abuse where victims experience threats, humiliation, or intimidating behaviors.
Anusiewicz et al^13^	2019	USA	Bullying	Workplace bullying, originally termed “mobbing,” is used interchangeably with workplace bullying (WPB).
Anusiewicz et al^18^	2021	USA	Bullying	Workplace bullying (WPB) involves any negative behavior exhibited by an individual or a group with perceived or actual power, repeatedly and persistently directed toward another individual who has difficulty defending themselves against the behavior.
Arnetz et al^16^	2019	USA	Bullying	Workplace bullying defined as repeated abusive mistreatment.
Beitz et al^14^	2021	USA	Bullying	Mobbing (or bullying) as experiencing at least 1 of the behaviors within the last 12 months and occurring at least weekly or monthly over the past 6 months.
Fry et al^17^	2021	USA	Bullying	Bullying is repeated and unwanted harmful actions that are intended to humiliate, offend, and cause distress in the target.
Vessey et al^21^	2010	USA	Bullying	Occurs when there is a perceived or actual power difference between the perpetrator and the target, leading to threats, intimidation, psychological distress, or other forms of harm.
Pfeifer and Vessey^20^	2017	USA	Bullying and lateral violence	Repeated, offensive, abusive, and intimidating behaviors that make the target feel upset, humiliated, vulnerable, or threatened, causing undue stress and reduced confidence.
Christensen et al^52^	2021	Australia	Contra power harassment	Harassment by nursing students is a growing phenomenon which is defined as the harassment of those in formal positions of power by those who are not.
Rosi et al^45^	2020	Italy	Horizontal violence	Described as a negative interaction or interpersonal conflict between two nurses of similar positions and hierarchical levels. This interaction may be psychological, verbal, or physical, and can be characterized by discrimination, prejudice, or lack of support.
McKenna et al^46^	2003	New Zealand	Horizontal violence	Most commonly takes the form of psychological harassment, which fosters hostility rather than physical aggression. This harassment includes verbal abuse, threats, intimidation, humiliation, excessive criticism, innuendo, exclusion, disinterest, discouragement, and withholding of information.
Dumont et al^53^	2012	USA	Horizontal violence	Behavior that humiliates, degrades, or otherwise indicates a lack of respect for the dignity and worth of an individual
Blackstock et al^30^	2022	Canada	Incivility	Incivility encompasses a range of negative behaviors such as name-calling, making rude comments, eye-rolling, and attacking a person’s integrity. Workplace incivility is characterized by behaviors that are repeated and recurring.
Laschinger et al^37^	2016	Canada	Incivility	Behaviors with an ambiguous intent to harm others and violate norms of courteous and respectful conduct.
Kousha et al^31^	2022	Iran	Incivility	Incivility is a dysfunctional interaction in the nursing workplace. Incivility is verbal and nonverbal behaviors that demean, reject, or exclude the person.
Abolfaz Vaghar^36^	2015	Iran	Incivility	Is a behavior of low intensity and ambiguous intent, which lacks mutual respect.
Park and Kang^12^	2023	Korea	Incivility	Multiple terms are used interchangeably with incivility, including horizontal violence, mobbing, and bullying.
Karacay and Oflaz^7^	2022	Turkey	Incivility	Defined as the rude and discourteous speech or act that aims to hurt one’s self-esteem by arousing suspicion about one’s abilities.
Authement^27^	2016	UK	Incivility	Incivility is also called bullying, lateral violence, and horizontal violence.
Clark et al ^32-34^	(2009, 2008, 2022)	USA	Incivility	Behaviors which often result in psychological or physiological distress for the people involved.
Gallo^59^	2012	USA	Incivility	Incivility is characterized by disrespect for others. It includes, but is not limited to, disrespectful behaviors, condescending speech or attitudes, academic dishonesty, bullying, and violent or potentially violent behaviors.
Hudgins et al^58^	2022	USA	Incivility	Violates the norms of mutual respect in the teaching–learning environment.
Hunt et al^57^	2012	USA	Incivility	The term “incivility” encompasses low-intensity behavior that lacks a clear intent to harm, but nevertheless violates social norms and can cause harm.
Kerber et al^38^	2015	USA	Incivility	Uncivil behavior has been characterized as rude, disrespectful, and intimidating.
Lampley et al^29^	2016	USA	Incivility	Incivility in nursing education is a serious global issue that negatively impacts student and teachers relationships as well as the educational process. Common terms used to describe this phenomenon include bullying, cyberbullying, lateral violence, violence, disruptive behavior, horizontal violence, misconduct, and mobbing.
McPherson and Buxton^35^	2019	USA	Incivility	Most of the literature agree that incivility breaches the workplace norms of respect and courtesy.
Palumbo^28^	2018	USA	Incivility	Encompasses lateral and horizontal violence or any other disruptive behavior or speech that negatively affects others.
Patel and Chrisman^49^	2020	USA	Incivility	Incivility as acting with disregard for others in the workplace.
Lamontagne^50^	2013	USA	Intimidation	Described as one or multiple unjust verbal statements by someone in authority that negatively impacts the target and bystanders.
Rittenmeyer et al^44^	2013	USA	Lateral/horizontal violence	Nurse-on-nurse aggression refers to destructive behavior among nurses directed at one another. Lateral or horizontal (L/H) violence involves demeaning and downgrading others through unkind words and cruel acts, often leading to diminished confidence and self-esteem in the targets.
Tong et al^48^	2017	Switzerland	Mobbing	As repeated, unreasonable behavior toward an employee, or group of employees that create a risk to health and safety.
daCruz et al^41^	2017	Brazil	Moral harassment	Moral harassment arises from psychic suffering.
Hagopian et al^42^	2019	Brazil	Moral harassment	A kind of perverse violence that is characterized by repeated behaviors that threaten the dignity or physical or psychological integrity of a person.
Hawkins et al^8^	2019	Australia	Negative workplace behavior	Negative workplace behavior was used throughout this review to provide a description of the bullying, harassment, horizontal violence, and incivility experienced by nurses.
Khalil^47^	2009	South Africa	Psychological violence	Bullying, verbal abuse, gossiping, marginalizations, public humiliation, and all forms of nonphysical behaviors that result in emotional discomfort for another person or colleague.
Gee and Copeland^40^	2023	USA	Shaming	Shaming is the action of expressing condemnation of a characteristic or behavior to an audience with the intention of invoking a shame response.
Yancey^39^	2021	USA	Shaming	The act of shaming may arise in many forms including bullying online shaming, cyberbullying, and mob shaming.
Thomas and Burk^43^	2009	Canada	Vertical violence but defined horizontal violence	Horizontal violence (also known as lateral violence) refers to abusive behaviors occurring between coworkers of similar status, such as staff nurses, within the workplace. It is a form of workplace violence.
Hamblin et al^51^	2015	USA	Worker to worker violence	Incivility has been defined as a low-intensity deviant behavior with an ambiguous intent to cause harm.
Agrawal et al^54^	2023	India	Workplace violence	Incidents where staff is abused, threatened, or assaulted at work and includes verbal abuses, threats as well as physical attacks.
Mamaghani et al^55^	2022 and 2023	Iran	Workplace violence	Verbal type and involved calling inappropriately, yelling, humiliating one another in the presence of the instructor, medical staff, or patients.
Al-Qadi^56^	2021	USA	Workplace violence	Workplace violence encompasses any act or threat of physical violence including harassment, intimidation, or other threatening disruptive behavior occurring at the work with the intent to abuse or injure the target.

#### Bullying

The concept of bullying in the workplace is a complex and multifaceted issue, often defined inconsistently across studies. Anusiewicz et al^13^ and Beitz et al^14^ include mobbing within their definitions of bullying, a perspective also supported by Park and Kang.^12^ Leong et al^11^ describes a subset of bullying behaviors that encompass horizontal or lateral violence, oppressed group behavior, incivility, harassment, counterproductive behavior, and aggression. Katrinli et al,^15^ Arnetz et al,^16^ Fry et al,^17^ Beitz et al,^14^ Anusiewicz et al,^18^ Park and Kang,^12^ Reknes et al,^19^ and Pfeifer and Vessey^20^ all emphasize the repetitive nature of bullying. Furthermore, Vessey et al^21^ and Anusiewicz et al^18^ highlight the importance of a power differential that must exist between the perpetrator and the target in defining bullying. While Malinauskiene,^22^ Strandmark et al,^23^ and O’Flynn-Magee et al ^24^ all refer to psychosocial impacts including isolation and exclusion.

Characteristics of bullying behaviors often include verbal abuse, threats, humiliation, or intimidation, with the perpetrator’s actions interfering with the target’s job performance.^15^ O’Flynn-Magee et al^24^ describe bullying as inappropriate conduct or comments that a person knew or reasonably ought to know would cause humiliation or intimidation to a worker or a student. Arnetz et al^16^ refer to bullying as abusive mistreatment, while Fry et al^17^ identify it as harmful actions intended to humiliate, offend, or cause distress. Lee et al^25^ includes both verbal and physical violence in their definition of bullying. Park and Kang^12^ describe bullying behaviors as harassing, offending, or socially excluding someone, or negatively affecting their work. Similarly, Malinauskiene et al^22^ and Pfeifer and Vessey^20^ include social isolation or exclusion, devaluation of work efforts, threats, derogatory comments, or other behaviors aimed at tormenting, wearing down, or frustrating the target as bullying actions. However, as Hartin et al^26^ noted in their review, there is no universally accepted definition of bullying in the literature.

#### Incivility

The concept of incivility is often conflated with bullying, lateral violence, and horizontal violence. Authement^27^ and Palumbo^28^ suggest that incivility is frequently referred to using these terms, a view echoed by Lampley et al,^29^ who list bullying, cyberbullying, lateral violence, disruptive behavior, and mobbing as common descriptors of incivility. Park et al^12^ also notes that terms such as horizontal violence, mobbing, and bullying are used interchangeably with incivility. Blackstock et al^30^ identify uncivil behaviors as including name-calling, rude comments, eye-rolling, and attacks on a person’s integrity. Kousha et al^31^ expand on this by including verbal and nonverbal behaviors that demean, reject, or exclude individuals. Clark et al ^32-34^ refer to the psychological and physiological distress caused by the behaviors, while the notion of respect, or the lack thereof, is frequently associated with incivility, as noted by McPherson and Buxton,^35^ Vagharseyyedin,^36^ Laschinger et al,^37^ and Kerber et al.^38^ Common descriptors of the behaviors associated with incivility are “disrespect”^59^ and “discourteous”^7^; however, authors also denote a range of behaviors including potentially violent behaviors.^39-61^

## Discussion

Despite bullying and incivility being the most common terms used to describe unwanted workplace behaviors, new terms such as shaming, cyber bullying, moral harassment, and contrapower have been more recently introduced. Shaming as defined by Yancey^39^ and Gee and Copeland^40^ includes reference to online modalities such as cyber bullying, but both authors suggest shaming requires an audience in order to evoke the shame response, be that virtual or actual. The two authors that refer to moral harassment, daCruz et al^41^ and Hogopian et al^42^ are both from Brazil and draw similarities with incivility whereby the definition includes reference to the target’s dignity and integrity. Contrapower harassment is another term more recently presented in the literature.^52^ This term can be likened to vertical,^43^ lateral,^20,44^ or horizontal violence^45-47,53^ through which positions of power differentiate the target and perpetrator, as described in the definitions of bullying. Contrapower harassment was specifically used to describe student harassment of faculty.^52^

The review noted that the severe emotional and psychological impact on targets and witnesses can lead to significant distress.^21, 32-34,42,45,46^ Feelings of humiliation, intimidation, and social exclusion have been highlighted as an intent and consequence of unwanted workplace behaviors.^15,46,48^ The review also noted unwanted workplace behaviors negatively impact the work environment, which leads to decreased job satisfaction and increased stress and desire to leave employment.^32-34,17,20^

It is unclear when or how nurses learn, accept, and model acts of unwanted workplace behaviors, but we know student nurses are also vulnerable to unwanted workplace behaviors despite not being considered part of the recognized workforce.^24,29^ There is evidence to suggest that student nurses are exposed to these behaviors during workplace learning^5,60^ and exhibit similar acts themselves participating in simulation learning and teaching activitites.^61^

Based on this review, it is clear there is a need for a universal term and definition of unwanted workplace behaviors. Authors such as Boyle and Wallis^62^ made a call in 2016, identifying the lack of consensus as a barrier to nursing researchers’ ability to compare and contrast perceptions and incidences of unwanted behaviors across disciplines and countries. Standardized terminology would improve recognition, reporting, and management of these behaviors, which in turn would facilitate better research, policy, and practical workplace interventions.^26^

Unwanted workplace behaviors involve a form of aggression, whether overt or subtle, and contribute to a hostile work environment. However, each behavior differs in their specific dynamics and manifestations. While these terms have been used to describe destructive behaviors that disrupt the workplace, it seems that the nuances in their definitions are what separates the application of terms.^49-61^

Each term addresses different forms and levels of severity of unwanted workplace behaviors. However, each behavior shares intent. The intent of the behaviors is to intimidate, humiliate, disrespect, and isolate.^15,46,47^ More attention is needed to understand the motivation of the behaviors rather than labeling of the unwanted workplace behaviors that remain undesirable and unsafe in nursing practice regardless of the terminology used to describe them.

### Limitations

Several potential limitations in the review methods may have influenced the findings. First, many of the studies relied on self-reported data, and this may result in under- or over-reporting of behaviors. While the review identified a wide range of terms, it did not assess the impact of these behaviors in specific cultural or organizational contexts, which may vary significantly across different healthcare settings. Despite including international studies, the heterogeneity of the studies in terms of settings, populations, and definitions of unwanted behaviors may limit the comparability of results. The review also predominantly included studies from high-income countries, which may not be generalizable to low- and middle-income settings where healthcare dynamics and workplace cultures differ significantly. The review included only original research studies which excluded possible relevant discussion and opinion pieces and unpublished or gray literature that could provide additional insights into the terminology used to describe unwanted workplace behaviors in nursing. The search included the term “workplace” which seemed to remove studies that focused on unwanted behaviors experienced from patients and family members, leaving the focus on nursing workforce.

## Conclusion

This scoping review highlights significant variability in the terminology used to describe unwanted workplace behaviors in nursing. Addressing this variability is essential for advancing research, policy, and practice. Clear, consistent definitions and a singular graded term will improve the recognition, reporting, and management of these behaviors, ultimately enhancing the work environment for nurses and the care they provide to patients. By establishing a standardized language, researchers can more accurately measure the prevalence and impact of these behaviors, leading to more robust and comparable data across studies. Policymakers can develop more targeted interventions and regulations to mitigate these behaviors, while healthcare organizations can implement more effective training and support systems for their staff. Ultimately, a unified approach to defining and addressing unwanted workplace behaviors will contribute to a more sustainable, safer and more productive healthcare environment, benefiting both healthcare workers and patients alike.
